# *Toxoplasma gondii* apicoplast-resident ferredoxin is an essential electron transfer protein for the MEP isoprenoid-biosynthetic pathway

**DOI:** 10.1016/j.jbc.2021.101468

**Published:** 2021-12-08

**Authors:** Stephanie Henkel, Nora Frohnecke, Deborah Maus, Malcolm J. McConville, Michael Laue, Martin Blume, Frank Seeber

**Affiliations:** 1 Mycotic and Parasitic Agents and Mycobacteria (FG16), Robert Koch Institute, Berlin, Germany; 2 Metabolism of Microbial Pathogens (NG2), Robert Koch Institute, Berlin, Germany; 3 Department of Biochemistry and Pharmacology, Bio21 Institute of Molecular Science and Biotechnology, University of Melbourne, Melbourne, Australia; 4 Advanced Light and Electron Microscopy (ZBS 4), Robert Koch Institute, Berlin, Germany

**Keywords:** Toxoplasma gondii, protein isoprenylation, parasite metabolism, ferredoxin, iron-sulfur protein, mevalonate, plastid, apicoplast, aTc, anhydrotetracycline, DMAPP, dimethylallyl diphosphate, DOXP, 1-deoxy-D-xylulose 5-phosphate, DXS, 1-deoxy-D-xylulose-5-phosphate synthase, EcFldA, *E. coli* flavodoxin A, FASII, type II fatty acid synthase, FBS, fetal bovine serum, Fd, ferredoxin, Fld, flavodoxin, FNR, Fd NADP^+^ reducatse, HFF, human foreskin fibroblasts, HMBPP, 1-hydroxy-2-methyl-2-butenyl 4-diphosphate, iΔ, inducible knock-down, IFA, Immunofluorescence assay, IPP, isopentenyl diphosphate, LipA, lipoic acid synthase A, MEcPP, 2-C-methyl-D-erythritol 2,4-cyclodiphosphate, MEP, 2C-methyl-D-erythritol 4-phosphate, MOI, multiplicity of infection, MVA, mevalonate, p.i., post-induction, PDH, pyruvate dehydrogenase, pt, plant-type, UPRT, uracil phosphoribosyltransferase

## Abstract

Apicomplexan parasites, such as *Toxoplasma gondii*, are unusual in that each cell contains a single apicoplast, a plastid-like organelle that compartmentalizes enzymes involved in the essential 2C-methyl-D-erythritol 4-phosphate pathway of isoprenoid biosynthesis. The last two enzymatic steps in this organellar pathway require electrons from a redox carrier. However, the small iron-sulfur cluster-containing protein ferredoxin, a likely candidate for this function, has not been investigated in this context. We show here that inducible knockdown of *T. gondii* ferredoxin results in progressive inhibition of growth and eventual parasite death. Surprisingly, this phenotype is not accompanied by ultrastructural changes in the apicoplast or overall cell morphology. The knockdown of ferredoxin was instead associated with a dramatic decrease in cellular levels of the last two metabolites in isoprenoid biosynthesis, 1-hydroxy-2-methyl-2-(E)- butenyl-4-pyrophosphate, and isomeric dimethylallyl pyrophosphate/isopentenyl pyrophosphate. Ferredoxin depletion was also observed to impair gliding motility, consistent with isoprenoid metabolites being important for dolichol biosynthesis, protein prenylation, and modification of other proteins involved in motility. Significantly, pharmacological inhibition of isoprenoid synthesis of the host cell exacerbated the impact of ferredoxin depletion on parasite replication, suggesting that the slow onset of parasite death after ferredoxin depletion is because of isoprenoid scavenging from the host cell and leading to partial compensation of the depleted parasite metabolites upon ferredoxin knockdown. Overall, these findings show that ferredoxin has an essential physiological function as an electron donor for the 2C-methyl-D-erythritol 4-phosphate pathway and is a potential drug target for apicomplexan parasites.

Isoprenoids, derived from the simple unsaturated hydrocarbon isoprene, are the largest and most diverse group of compounds found in nature, with more than 23,000 described structures (1). Besides their role as secondary metabolites in plants, complex isoprenoids are involved in a number of important cellular processes such as signaling, protein modifications (prenylation and glycosylation), cofactor synthesis (ubiquinone), and tRNA modifications. The two cellular building blocks that are at the base of this diversity are isopentenyl diphosphate (IPP) and its isomer dimethylallyl diphosphate (DMAPP). Convergent evolution has resulted in two independent and principally different pathways for their synthesis, starting from different precursors and using unrelated enzymes (see Fig. S1). The so-called mevalonate (MVA) pathway starts with two molecules of acetyl-CoA and requires six enzymes for IPP synthesis (with three known variations in archaea), whereas the 2C-methyl-D-erythritol 4-phosphate (MEP) pathway uses seven enzymes to convert D-glyceraldehyde 3-phosphate and pyruvate to IPP (2). The distribution of the two pathways in different organisms is diverse and complex, with most eubacteria using only the MEP pathway whereas most nonplant eukaryotes rely on the cytosolic MVA pathway for IPP synthesis. Notably, photosynthetic eukaryotes harbor both pathways, with the MVA pathway in the cytosol and, because of the cyanobacterial origin of the plastid, the MEP pathway in this organelle (3).

Eukaryotic parasites belonging to the phylum Apicomplexa, including *Plasmodium falciparum*, the causative agent of human malaria, use the MEP pathway. Early studies showed that this pathway is inhibited by the drug fosmidomycin (4) and that key enzymes in this pathways are localized in the apicoplast, a single plastid-derived organelle surrounded by four membranes (5, 6), which had been acquired by secondary endosymbiosis of a red alga (7). The Apicomplexa lack MVA pathway genes, suggesting that these protists are entirely dependent on the MEP pathway for *de novo* isoprenoid synthesis.

The absence of the MEP pathway in the mammalian host has raised the possibility that isoprenoid biosynthesis is a drug target in *P. falciparum* and other Apicomplexa, such as *Toxoplasma gondii* (8, 9). *T. gondii* causes toxoplasmosis in animals and humans and is one of the most prevalent protozoan infections worldwide (10). As in *P. falciparum*, the enzymes involved in isoprenoid synthesis in *T. gondii* are nuclear encoded but contain N-terminal topogenic targeting signals and are localized to the apicoplast (9, 11). Notably, studies in *Escherichia coli* suggest that the MEP pathway also requires the presence of a flavodoxin to provide electrons for the last two enzymes, IspG (GcpE) (12) and IspH (LytB) (13, 14). In particular, *E. coli* flavodoxin A (FldA) is essential (15), whereas its loss can be rescued by a transgenic MVA bypass system (16). Conversely, overexpression of FldA and the bacterial ferredoxin/flavodoxin (Fd/Fld) reductase in *E. coli* substantially increased isoprenoid production (17). Interestingly, the genomes of apicompexan protists do not encode flavodoxin-like redox proteins. Instead, they possess the small acidic [2Fe-2S] plant-type ferredoxin, which together with the pt ferredoxin-NADP(H) reductase (FNR) constitute an active redox system in the apicoplast (18, 19). Previously, it was shown that pt ferredoxin from *P. falciparum* physically interacts with PfIspH and supports its enzymatic activity *in vitro* (20). However, whether Fd presents the sole reducing force in plastids and related organelles remains unknown in the Apicomplexa, plants, and algae.

Here, we show by conditional depletion of TgFd (TGME49_215070) that the gene is essential in *T. gondii* and that the protein fulfills a critical function in the MEP pathway’s last two enzymatic steps. The inducible knock-down strain is devoid of IPP/DMAPP, and this deficiency is reflected by several impaired pathways that depend on isoprenoid biosynthesis (see Fig. 1).

**Figure 1:**
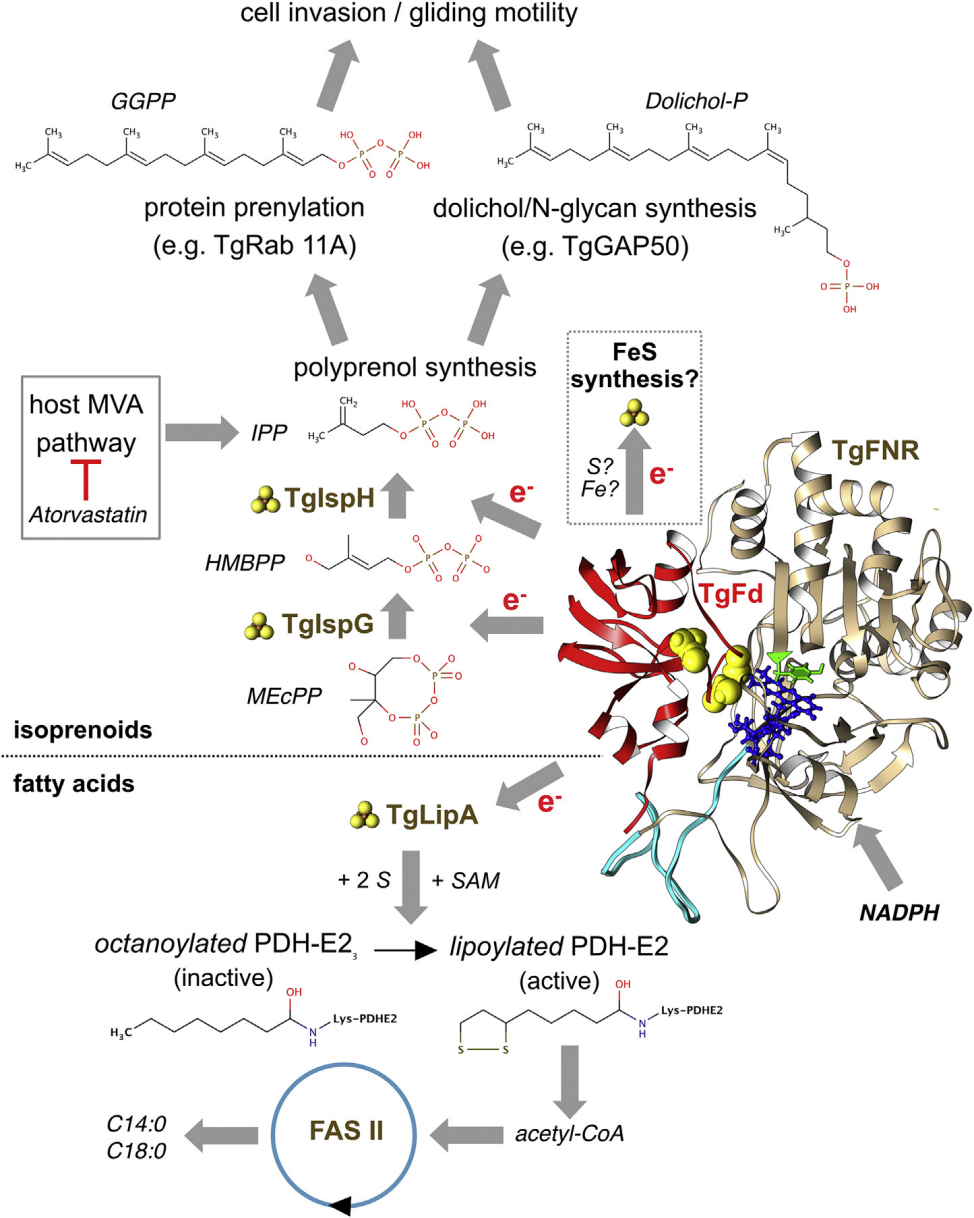
**Ferredoxin’s role in the metabolism of the apicoplast.** Metabolites/compounds are in *italics*; enzymes in *brown*. The model of the TgFd–TgFNR complex is derived from (88). The electron flow from TgFd to TgIspG/TgIspH and TgLipA (marked as Fe-S-containing proteins by ball-and-stick images) starts from NADPH *via* TgFNR. The possible involvement of TgFd as reductant for sulfur and/or iron in the synthesis of Fe-S clusters (8, 9) is indicated, as well as scavenging of host IPP and its synthesis inhibition by atorvastatin. FASII, fatty acid synthesis type II; Fd, ferredoxin; FNR, Fd NADP^+^ reductase; HMBPP, 1-hydroxy-2-methyl-2-butenyl 4-diphosphate; IPP, isopentenyl diphosphate; LipA, lipoic acid synthase; MEcPP, 2-C-methyl-D-erythritol 2,4-cyclodiphosphate; MVA, mevalonate; PDH, pyruvate dehydrogenase; SAM, S-adenosyl methionine.

## Results

### Conditional down-regulation of ferredoxin expression and its influence on the survival of *T. gondii*

**Figure 2:**
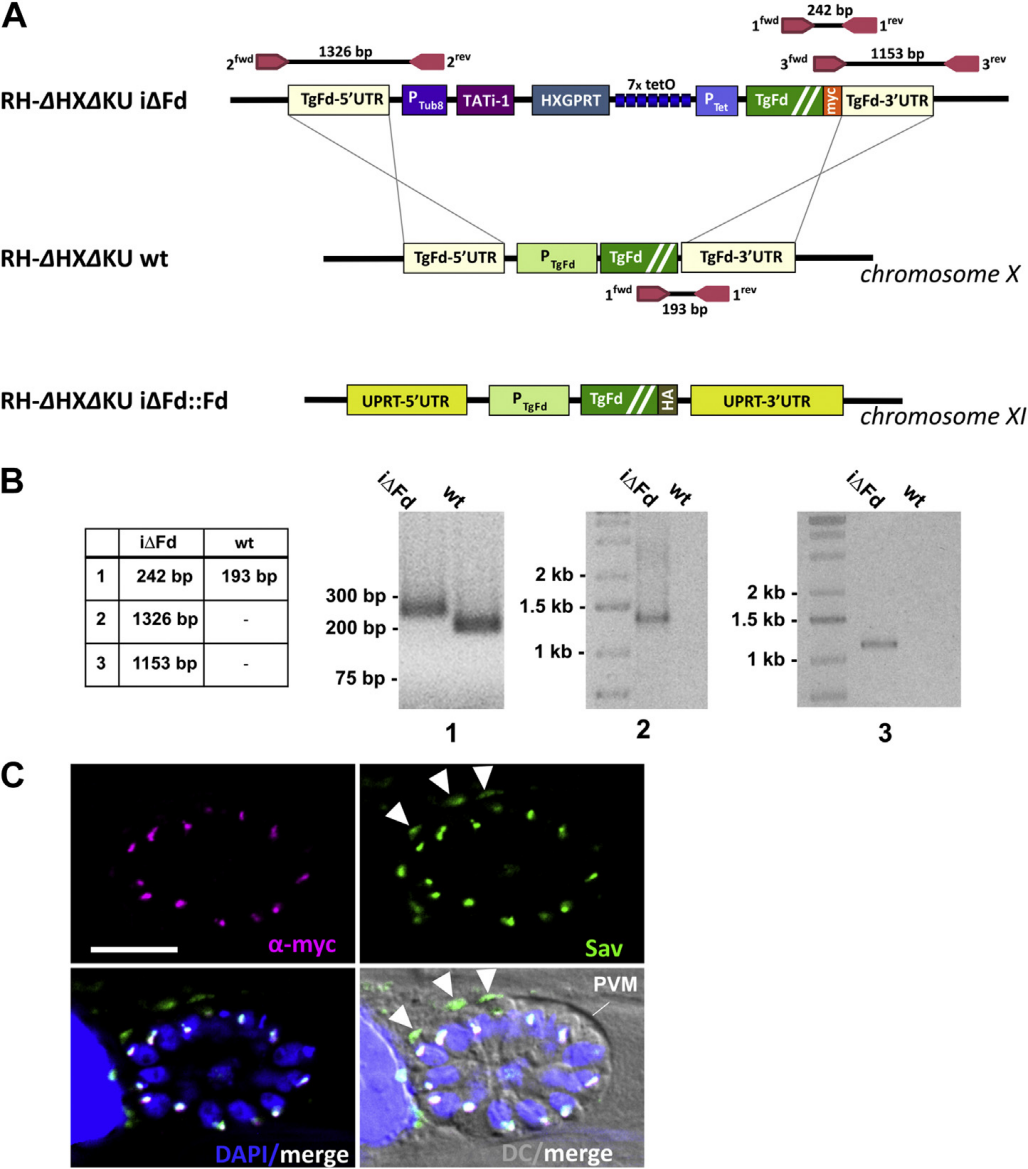
**Generation and characterization of iΔFd strain.***A*, scheme of genetic constructs used to generate iΔFd strain by replacement of the endogenous gene locus through double-crossover homologous recombination. The construct for complementation *via* insertion at the UPRT locus is shown at the *bottom*. *B*, analytical PCR on iΔFd genomic DNA that confirms proper integration of the inducible construct and replacement of the endogenous TgFd gene. WT DNA served as control. Primer combinations and expected amplicon sizes are given in *A*. *C*, *top left*, TgFd_myc_; *top right*, streptavidin Alexa Fluor 488; *bottom left*, merged DNA stain (DAPI) with *green* and *magenta* (false color) fluorescent images, with colocalized apicoplast signal appearing *white* (all three signals). The scale bar represents 10 μm. *Arrowheads* mark the biotin signal from biotinylated proteins likely located in the host mitochondria, which adhere to the PVM in strain RH. Fd, ferredoxin; iΔ, inducible knock-down; UPRT, uracil phosphoribosyltransferase.

Assuming that TgFd would be essential (based on a negative phenotype score of −4.35; (21)), we used an inducible knock-down (iΔ) approach based on the previously described tetracycline-inducible transactivator system (22, 23). In this system, the endogenous single copy of TgFd was replaced in one step with a myc-tagged copy (TgFd_myc_) by double cross-over homologous recombination (see Fig. 2*A* for the replacement and complementation strategy). As shown in Figure 2*B*, analytical PCR experiments verified that the targeted endogenous TgFd locus had been replaced with the construct, and the resulting clone was termed iΔFd (for inducible knock-down of TgFd).

Immunofluorescence assays (IFA) using an anti-myc antibody showed expression of TgFd_myc_ under normal cell culture conditions exclusively in the apicoplast, confirmed by costaining with fluorescent streptavidin which gives a strong signal with biotin-bearing acetyl-CoA carboxylase in this organelle (24) and to some extent also with the host’s biotinylated carboxylases in the mitochondrion (25) (Fig. 2*C*).

**Figure 3:**
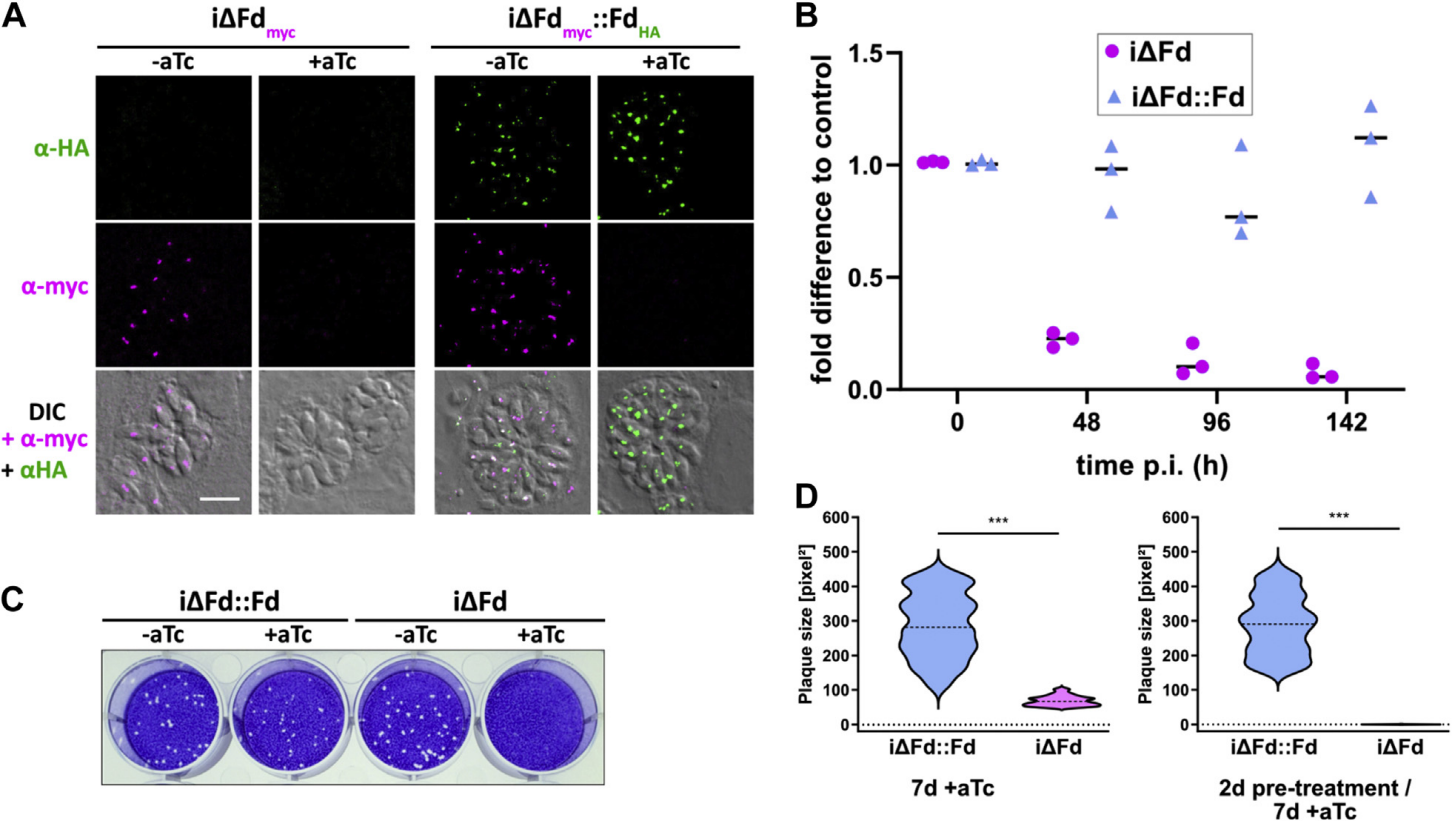
**Growth phenotype of iΔFd and its characterization.***A*, IFA of depleted and complemented iΔFd strain with antibodies against their epitope tags in dependence on aTc treatment. The scale bar represents 10 μm. *B*, RT-qPCR analysis of TgFd transcripts over 142 h after aTc induction of iΔFd and iΔFd::Fd strains. The fold difference compared with housekeeping gene actin is indicated. *C*, plaque assay after 7 days p.i. *D*, quantification of plaque sizes after 7 days, either without (*left*) or with 48 h of aTc pretreatment (*right*). ∗∗∗*p* ≤ 0.001; One-way ANOVA with Kruskal–Wallis test. aTc, anhydrotetracycline; Fd, ferredoxin; iΔ, inducible knock-down; IFA, immunofluorescence assay; p.i. postinduction.

Upon anhydrotetracycline (aTc) treatment of iΔFd, the expected disappearance of TgFd_myc_ was observed by IFA (Fig. 3*A*). Complete loss of IFA signal occurred after 48 h. TgFd transcript detected by RT-qPCR was greatly diminished by 48 h after kd induction and almost undetectable at 142 h postinduction (p.i.) (Fig. 3*B*). Interestingly, the initial growth of iΔFd parasites under inducing and noninducing conditions were similar in bulk culture (*i.e.*, passage of egressed parasites after circa 48 h). However, the growth of iΔFd parasites under inducing conditions was severely reduced in plaque assays over 6 to 7 days. The latter records several lytic cycles of tachyzoite growth (*i.e.*, invasion, replication, egress, and reinvasion) (Fig. 3, *C* and *D*). Preincubation of iΔFd for 48 h with aTc (to deplete the cells of TgFd) resulted in no plaques at all in a 7-days assay (Fig. 3*D*).

To rule out that the growth arrest was because of off-target effects of aTc, we complemented iΔFd with a genomic copy of TgFd under its own promoter on the plasmid pUPRT-HA (26). It includes 500 bp upstream of the start codon of TgFd and its entire intron-containing coding sequence in frame with a C-terminal HA tag, and GRA2-3′UTR (Fig. 2*A*). This construct is embedded by 5′- and 3′-UTRs of uracil phosphoribosyltransferase (UPRT), a nonessential gene, the disruption of which by homologous recombination allows for the selection of FUDR resistance. A single clone was isolated (iΔFd::Fd), and its growth in aTc-containing medium was compared with iΔFd in a plaque assay. In the absence of aTc, the two strains showed similar plaque numbers and sizes (Fig. 3, *B* and *C*), whereas iΔFd gave rise to only very few and barely visible small plaques when aTc was included in the medium, while iΔFd::Fd retained the capacity to induce plaques. Of note, our initial attempts to interfere with the ferredoxin redox system by using a previously identified single point mutant of the reductase TgFNR, which is enzymatically inactive and shows a tenfold increase in its affinity for TgFd (27), as a transdominant mutant did not result in an apparent growth defect (see Fig. S2 and associated discussion).

**Figure 4:**
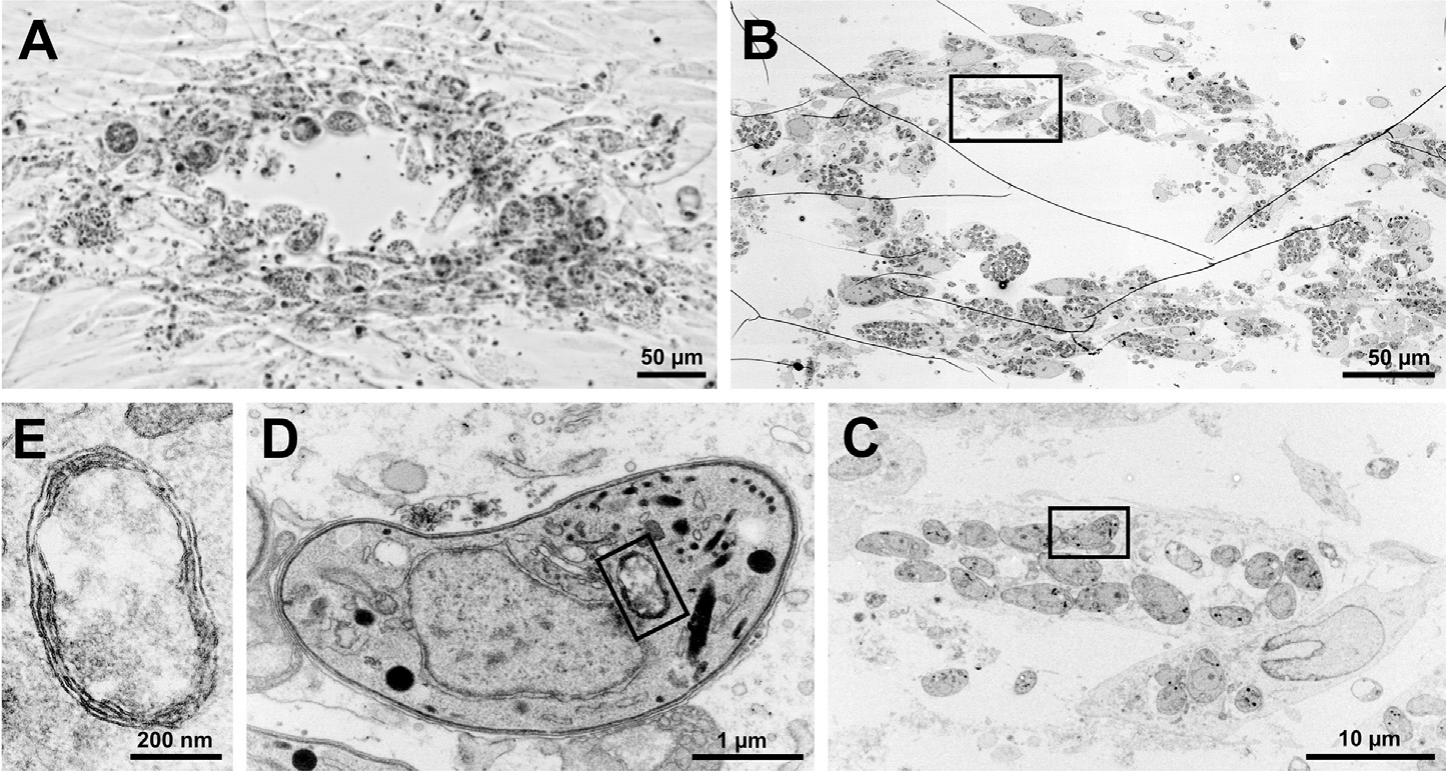
**Correlative light and electron microscopy of a single iΔFd plaque observable after 7 days of aTc treatment.***A*, light microscopic image of plaque. *B*, corresponding EM image of *A*. *C*–*E*, higher magnifications of boxed region from *B*, with ultrastructural detail of the four membranes of the apicoplast visible in *E*. Fd, ferredoxin; iΔ, inducible knock-down.

To observe possible ultrastructural changes caused by the knockdown of TgFd, we used correlative light and electron microscopy of parasites within plaques from aTc-treated iΔFd cultures (27). No apparent morphological changes could be observed when individual tachyzoites within tiny plaques of aTc-treated iΔFd cultures were compared with iΔFd::Fd controls (Fig. 4). In particular, TgFd depletion, even after 7 days, does not result in loss of the apicoplast. This is different from other gene depletion studies in *T. gondii*, for example, of Autophagy-Related Proteins ATG8 and ATG18, respectively, which resulted in delayed death and loss of the apicoplast within 4 to 5 days (28, 29) and ultrastructural changes in the organelle’s morphology after 24 h of aTc treatment (29). A similar rapid loss of apicoplasts upon drug treatment has been described recently (30).

### TgFd depletion results in disappearance of the two terminal MEP pathway metabolites

**Figure 5:**
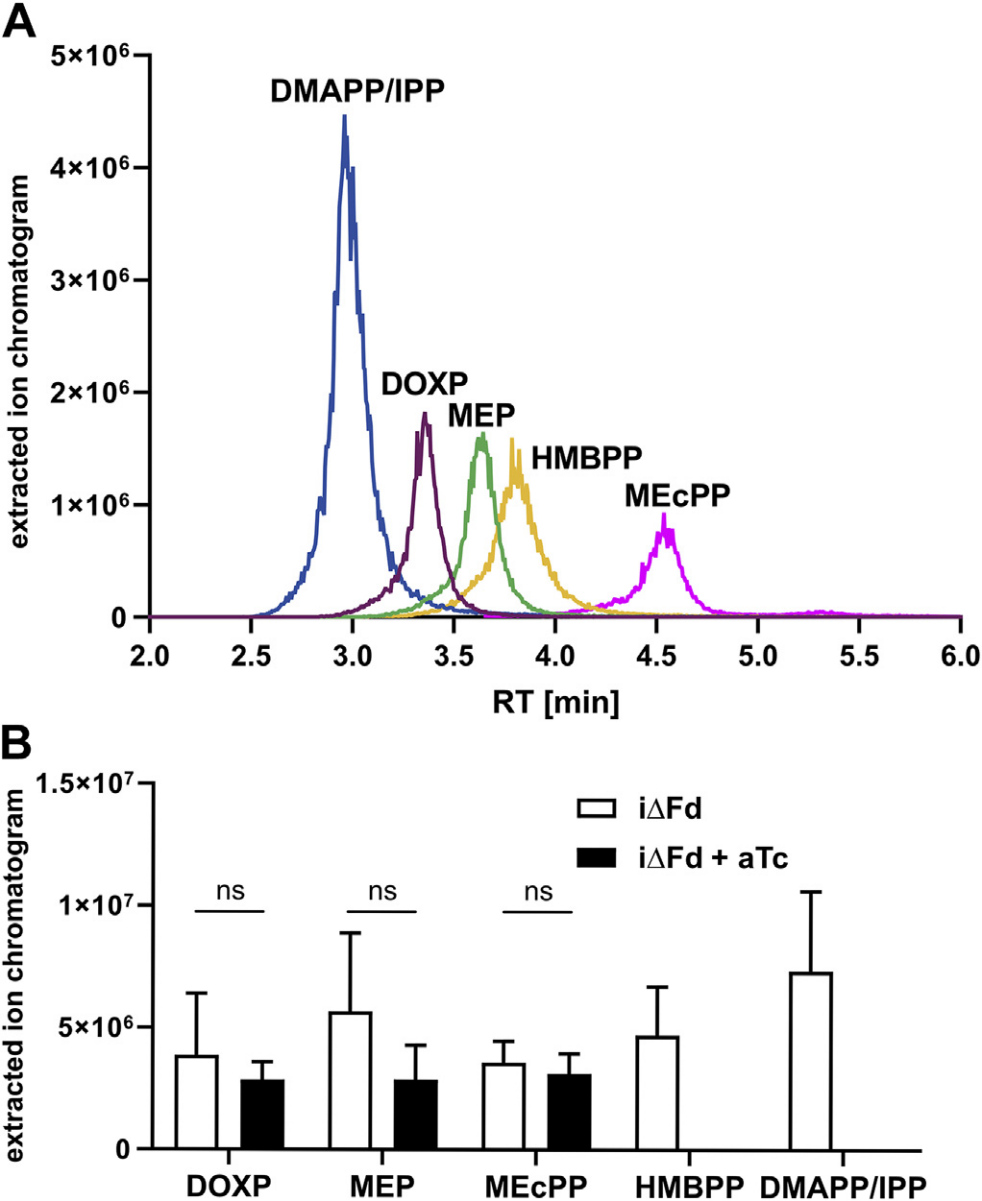
**Determination of MEP pathway metabolites in iΔFd::Fd parasites.***A*, retention time determination of MEP pathway standards DMAPP/IPP at 3 min, DOXP at 3.4 min, MEP at 3.7 min, HMBPP at 3.8 min, and MEcPP at 4.5 min. All standards were used at 100 nM. *B*, abundance of MEP pathway metabolites in iΔFd parasites in absence (*i.e.*, only vehicle) and presence of aTc analyzed by targeted LC-MS. The chromatographic peak area indicates the abundance of the specific metabolite after 96 h treatment. The bars indicate the mean ± SD of three independent biological replicates. Statistical analysis was performed with two-way ANOVA with Bonferroni’s correction for multiple testing. Because HMBPP and DMAPP/IPP were below detection limit in iΔFd + aTc samples, no *p*-value was calculated for those. ns = non-significant (*p* ≥ 0.05). aTc, anhydrotetracycline; DMAPP, dimethylallyl diphosphate; DOXP, 1-deoxy-D-xylulose-5-phosphate synthase; Fd, ferredoxin; HMBPP, 1-hydroxy-2-methyl-2-butenyl 4-diphosphate; iΔ, inducible knock-down; IPP, isopentenyl diphosphate; MEcPP, 2-C-methyl-D-erythritol 2,4-cyclodiphosphate; MEP, 2C-methyl-D-erythritol 4-phosphate.

To determine the consequences of a TgFd knock-down on the MEP pathway intermediates, we adapted and optimized published LC-MS protocols (31, 32) for use in *T. gondii*. This allowed us to separate and detect 1-deoxy-D-xylulose 5-phosphate (DOXP), MEP, 1-hydroxy-2-methyl-2-butenyl 4-diphosphate (HMBPP), and 2-C-methyl-D-erythritol 2,4-cyclodiphosphate (MEcPP) as individual chromatographic peaks, as well as the isomeric DMAPP and IPP as one peak (Fig. 5*A*). When we analyzed 1∗10^9^ tachyzoites per experiment of iΔFd parasites cultivated in the continuous presence of either aTc or vehicle alone for three generations (96 h), we observed comparable amounts of early intermediates in this pathway (DOXP, MEP, and MEcPP) in both cell pellets, whereas the products of TgIspG and TgIspH, HMAPP and IPP, respectively, were only detected in uninduced parasites (Fig. 5*B*). These analyses strongly suggest that TgFd is required for HMBPP and IPP production in the apicoplast.

### Host MVA pathway influences growth phenotype of iΔFd parasites

**Figure 6:**
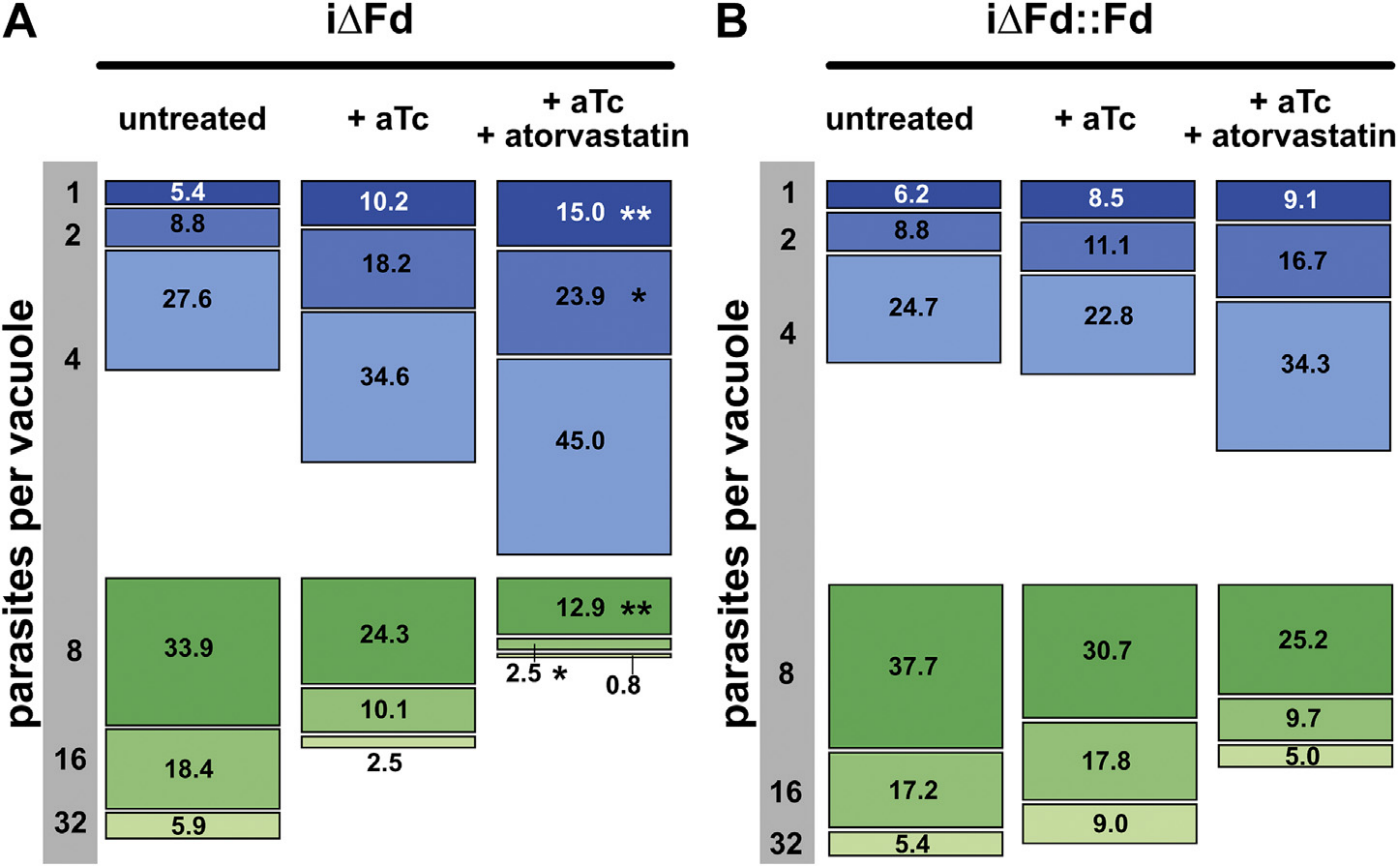
**Atorvastatin inhibition of host isoprenoid synthesis and its impact on parasite replication upon TgFd depletion.** Atorvastatin treatment at 13 μM was for 126 h. The number of vacuoles with one up to 32 parasites of each condition (*A*, iΔFd and *B*, iΔFd::Fd) are expressed as mean percentages of all vacuoles of three biological replicates, summing-up to 100%. ∗∗*p* ≤ 0.01; ∗*p* ≤ 0.05; pairwise comparison of the means against the untreated controls as reference were done using One-way ANOVA. aTc, anhydrotetracycline; Fd, ferredoxin; iΔ, inducible knock-down.

*T. gondii* are known to scavenge a wide range of carbon sources and essential nutrients from the host cell (33), including isoprenoids (34). We therefore evaluated whether inhibition of the host MVA pathway would exacerbate the loss of growth defect observed in parasites lacking TgFd. The infected host cells were treated with the HMG-CoA reductase inhibitor atorvastatin and the number of vacuoles containing from 1 to 32 parasites assessed. As shown in Figure 6, aTc treatment alone for 126 h of strain iΔFd::Fd did not lead to obvious growth inhibition, expressed as percentage of vacuoles containing the respective numbers of parasites. This is in contrast to strain iΔFd where the depletion of TgFd over this time period led to a shift in the number of parasites per vacuole, with a clear trend of fewer vacuoles with 8 to 32 tachyzoites and more with only 1 to 4 parasites/vacuole. However, when host isoprenoid synthesis was inhibited with 13 μM atorvastatin during the incubation period, growth inhibition of iΔFd was significant (Fig. 6). Interestingly, atorvastatin also induced a trend toward slowed-down intracellular replication of iΔFd::Fd parasites, consistent with previous data (34). Taken together, these experiments indicate that in both strains, host isoprenoid biosynthesis contributes to the growth of the parasites, but that iΔFd tachyzoites are significantly more affected by the insufficient amounts of IPP/DMAPP or their polymers it is able to scavenge from the host.

### Influence of Fd depletion on MEP pathway gene transcription

**Figure 7:**
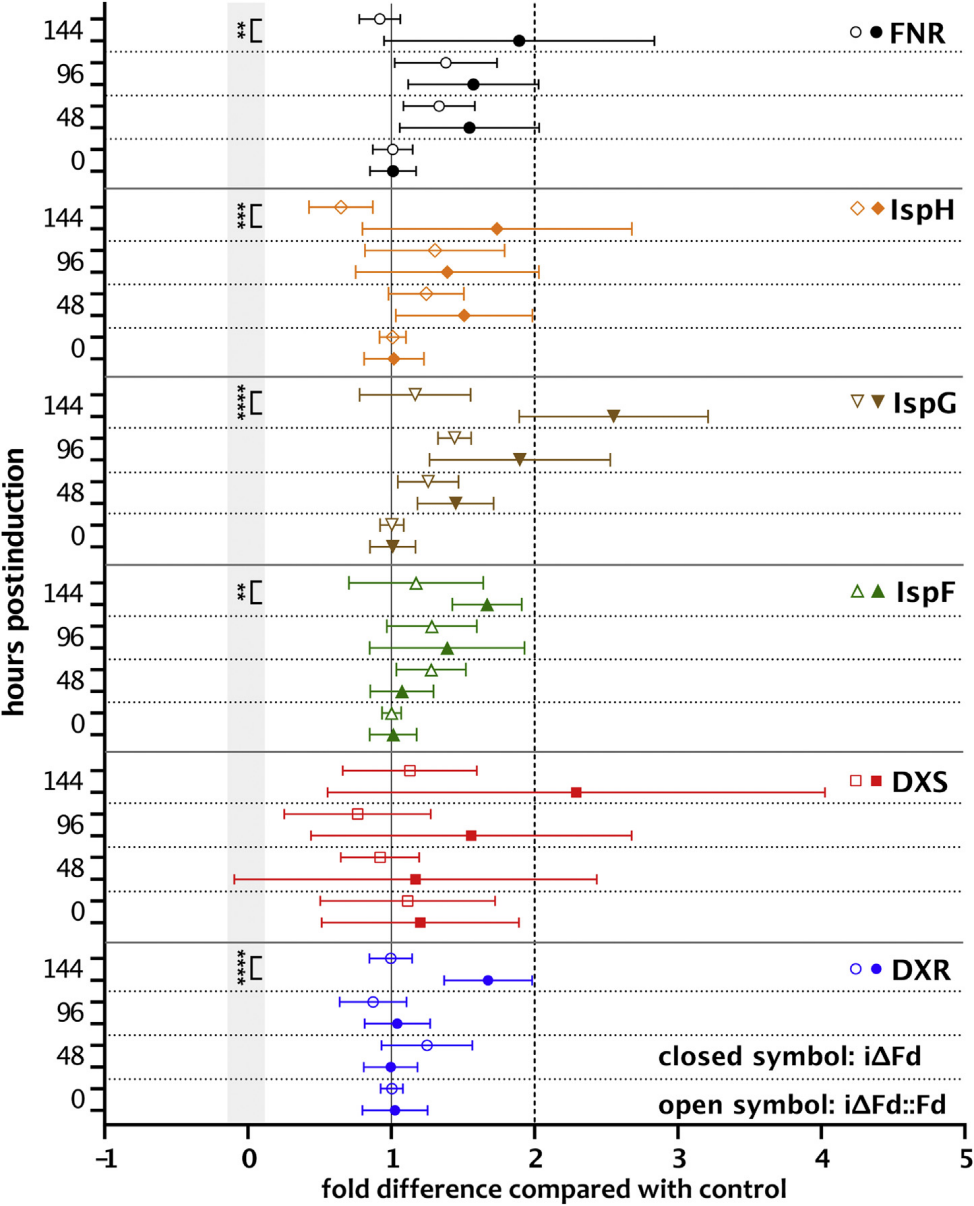
**Transcriptomic analysis of several MEP genes of iΔFd and iΔFd::Fd in dependence of aTc treatment *via* RT-qPCR.** Shown are the mean ± SD of fold difference compared with the housekeeping gene actin. ∗∗∗∗*p* ≤ 0.0001; ∗∗∗*p* ≤ 0.001; ∗∗*p* ≤ 0.01; unpaired Mann–Whitney U test (*shaded gray*). aTc, anhydrotetracycline; Fd, ferredoxin; FNR, Fd NADP reductase; iΔ, inducible knock-down; MEP, 2C-methyl-D-erythritol 4-phosphate.

The iΔFd strain provided an opportunity to analyze whether transcriptional regulation of genes of the MEP pathway is increased when key intermediates are depleted. We therefore analyzed the transcript levels of TgDXS, TgDXR, TgIspF, TgIspG and TgIspH, and TgFNR by RT-qPCR and compared iΔFd *versus* iΔFd::Fd parasites at different time points p.i. (Fig. 7). Considerable fluctuations in transcript levels were observed between replicate cultures, in particular for TgDXS. Overall, isoprenoid starvation only resulted in marginal (<2-fold) changes in the overall levels of expression of individual mRNA abundance after 144 h p.i. in iΔFd parasites compared with the complemented strain at the respective time point. These data indicate that, compared with other prokaryotes and eukaryotes, there is minimal transcriptional control on the genes in this pathway in response to depletion of late intermediates.

### Fd depletion influences gliding motility

**Figure 8:**
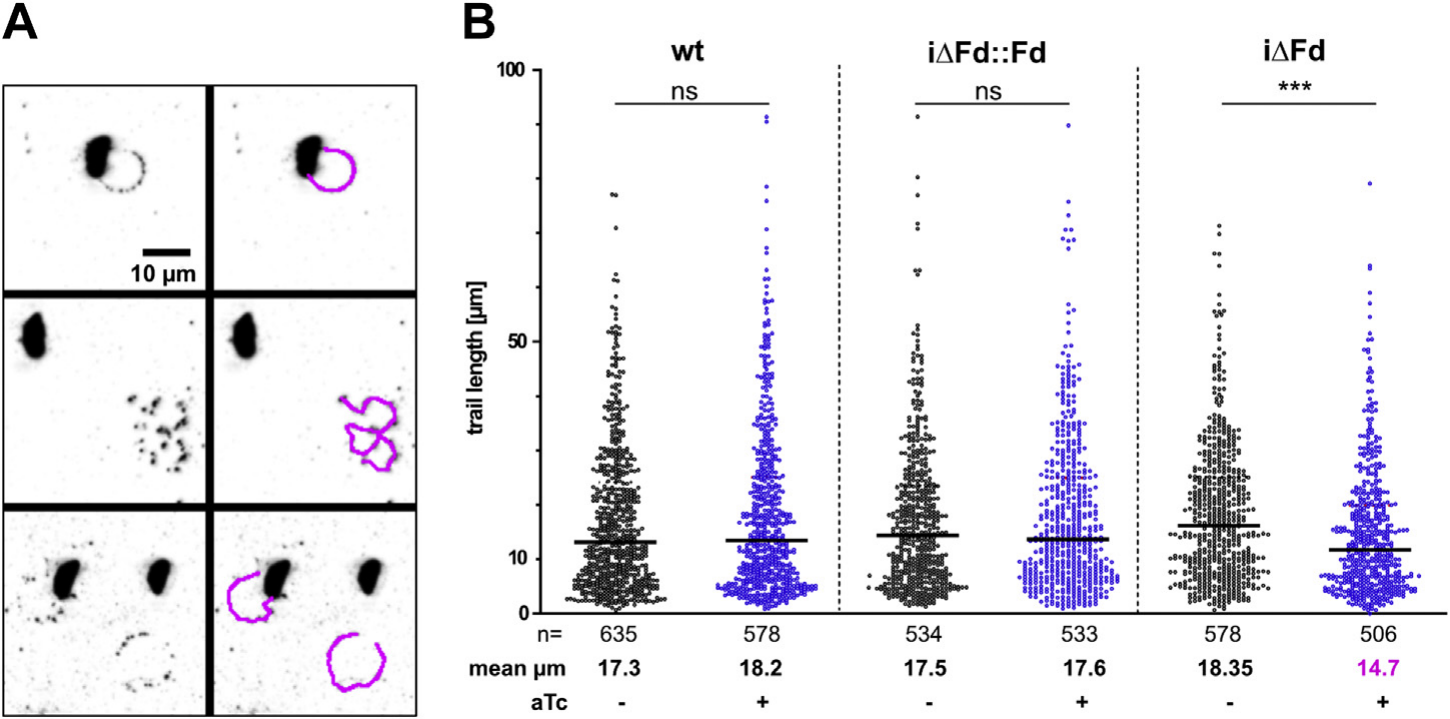
**Measurement of gliding motility impairment of iΔFd strain in comparison with controls.***A*, examples of fluorescent images (*inverted*) of trails (*left*) and their tracing by NeuronJ (*right*). *B*, individual measurements of trail lengths. The *horizontal lines* indicate medians. ∗∗∗∗*p* ≤ 0.0001; two-tailed Mann–Whitney U test. aTc, anhydrotetracycline; Fd, ferredoxin; iΔ, inducible knock-down.

It has been reported that inhibition of N-glycosylation greatly impairs gliding motility of tachyzoites (35). N-glycan precursors are assembled on dolichol-pyrophosphate in the ER and are thus directly dependent on IPP/DMAPP precursors (8). As a functional readout for consequences of IPP reduction, we therefore evaluated whether TgFd depletion would result in reduced gliding motility. This can be quantified by measuring the lengths of trails that extracellular tachyzoites leave behind on a protein-covered glass slide (36). The trails contain shed surface antigen 1, which can be visualized by antibody staining (Fig. 8*A*). Based on the blinded measurements of >500 trails per condition and strain, we observed a small but statistically significant decrease in median trail length of circa 27% between uninduced and iΔFd tachyzoites kept for 4 days in the presence of aTc, whereas aTc treatment neither had an effect on WT nor on iΔFd::Fd trail length and thus motility (Fig. 8*B*). We conclude that TgFd depletion affects the parasite’s gliding mobility to some extent.

### Fd depletion affects fatty acid synthesis in the apicoplast

**Figure 9:**
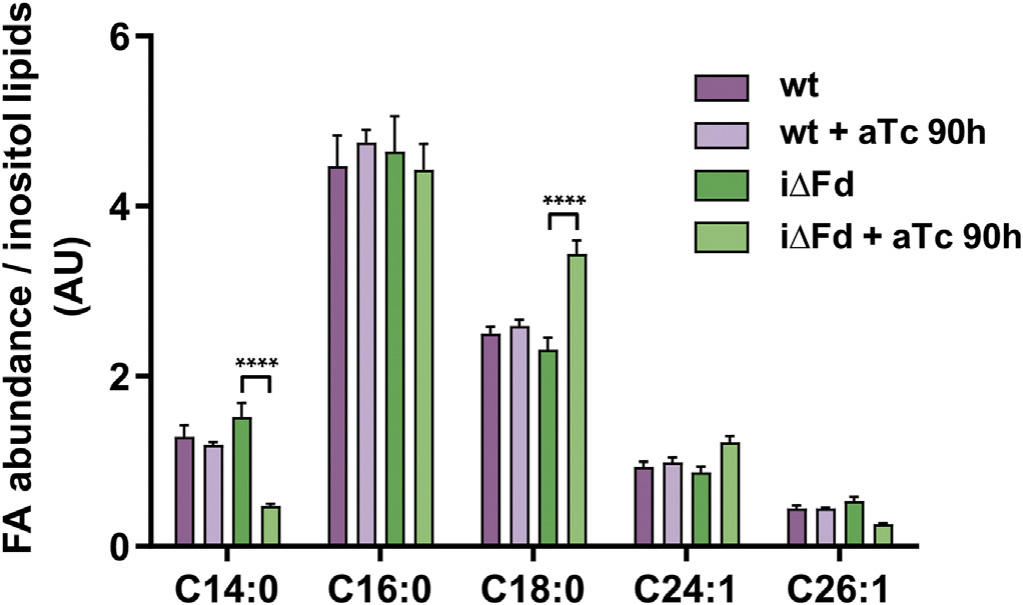
**Abundance of fatty acids extracted after TgFd depletion compared with controls according to LC-MS analysis.** ∗∗∗∗*p* ≤ 0.0001; two-way ANOVA with posthoc Bonferroni correction. aTc, anhydrotetracycline; Fd, ferredoxin; iΔ, inducible knock-down.

The *T. gondii* apicoplast harbors a pyruvate dehydrogenase (PDH) complex that provides acetyl-CoA for a type II fatty acid synthase pathway (FASII) (9). The E2 subunit of the apicoplast PDH contains a lipoic acid prosthetic group, which is synthesized *de novo* by and apicoplast-located lipoic acid synthase (LipA) (Fig. 1). LipA belongs to the group of so-called radical SAM enzymes (37) and requires a redox system for the generation of an adenosyl radical, involved in lipoate synthesis. Because TgFd was shown previously to physically interact with TgLipA (38), we investigated by GC-MS whether ^13^C glucose-labeling of iΔFd would show any changes in fatty acid composition compared with wt cells (Fig. 9). After 90 h of growth in the presence of aTc, a significant reduction of C14:0 and an increase in C18:0 fatty acids, respectively, was observed (Fig. 9). These data are very similar to those reported recently by Krishnan *et al.* (39) for a strain deleted in the FASII enoyl-CoA hydratase enzyme TgFabZ and provide evidence for TgFd’s expected role as an electron donor for TgLipA and thus its indirect involvement in FASII.

## Discussion

Isoprenoid biosynthesis plays a pivotal role in the metabolism of apicomplexan parasites, as suggested by fatal gene deletions of TgDXR and TgIspH in *T. gondii* (40) and PfDXR in *P. falciparum* (41), respectively. Our study is the first to show that Fd is also required for isoprenoid synthesis *in vivo*. A role of apicoplast-resident Fd in this pathway had been suggested earlier by *in vitro* studies with purified recombinant proteins from *P. falciparum* (PfIspH, PfFd, and PfFNR), thereby reconstituting the electron transport chain from NADPH/PfFNR to PfFd, resulting in the generation of IPP from HMBPP (20). That report also provided evidence for the physical interaction of PfFd and PfIspH, a prerequisite for electron transfer. Comparable results were reported for IspG and pt-Fd from the cyanobacterium *Thermosynechococcus elongatus* (42). However, whether this role of pt-Fd is also important *in vivo* has, to our knowledge, not been investigated in any system, that is, neither in plants or algae, where the MEP pathway is operating either alone or in parallel to the cytosolic MVA pathway.

Our data show that the TgFd-redox system is crucial by providing electrons to the two terminal enzymes, TgIspG and TgIspH. Conditional down-regulation of TgFd results in the depletion below detection limit of their respective products HMAPP and IPP (Fig. 1). In *E. coli*, the MEP pathway has been extensively studied, not the least due its industrial value for bacterial production of isoprenoids (43, 44), which includes the antimalarial compound artemisinin. However, in *E. coli*, a flavodoxin (EcFldA) acts as the redox partner, rather than a mitochondrial-type ferredoxin (EcFd). EcFldA is an essential gene (16, 45), but its deletion can be complemented by a bypass system. This is based on the terminal part of the MVA pathway, whereby externally added mevalonate is ultimately converted into IPP/DMAPP (16). Notably, EcFd is essential in the context of iron-sulfur cluster biosynthesis when the respective so-called SUF system is deleted (46) and again complementation requires a MVA-bypass system. The reason for this lies in the fact that both, IspG and IspH, are 4Fe-4S-containing enzymes and thus inhibition of cluster generation by EcFd deletion will render *E. coli* an isoprenoid auxotroph. Interestingly, the apicoplast also possesses a SUF-like ISC biosynthesis pathway (47) but no flavodoxin or other small electron donor, raising the possibility that TgFd might in addition be involved directly or indirectly in their 4Fe-4S cluster synthesis (48, 49). The extent to which TgFd is required for the activity of TglspG and TgIspH *versus* 4Fe-4S synthesis could be addressed by implementation of a MVA-based bypass approach, as was recently described in *P. falciparum* (41). IPP supplementation to the culture medium is not possible in *T. gondii* (unlike in *P. falciparum*) because the highly charged IPP and derivatives thereof (like fosmidomycin) are unable to reach the parasite cytosol (40, 50). Interestingly, DMAPP could not complement *P. falciparum* cultures whereas IPP can (51).

Metabolic engineering efforts in *E. coli* have provided insights into the necessity to fine-balance the levels of the individual metabolites of the MEP pathway, which might otherwise result in impaired cell growth due to their individual toxicity upon overproduction (52, 53, 54). Because the accumulation of pathway-specific metabolites cannot be counterbalanced by catabolic consumption, bacterial cells efflux superfluous DXP, MEP and in particular MEcPP, presumably through ABC-type transporters, as recently reported (53, 55, 56). Interestingly, under certain conditions IPP efflux by the ABC-type transporter ABCA1 has also been described in human γδ T cells (57). Noteworthy, MEcPP crosstalk between plant plastids and nucleus is well known, indicating that this metabolite is able to leave this organelle (58), but specific transporters have not been described so far (59). Whether such transport mechanisms out of the apicoplast and even out of the parasite cell exists in *T. gondii* is unknown. If so, it could explain why we did not observe higher MEcPP levels in iΔFd and, at the same time, resulted in only modest transcriptional regulation. In this respect, it is also important to note that IPP and DMAPP have been reported to inhibit plant DXS (1-deoxy-D-xylulose-5-phosphate synthase) activity by outcompeting the enzyme’s cofactor TPP (60). However, given that we observed no changes of metabolite abundance upstream of HMBPP, such TgDXS inhibition as an additional inhibitory factor contributing to iΔFd death is unlikely.

In *P. falciparum*, sublethal fosmidomycin treatment was used to assess the consequences of MEP pathway inhibition on the pathway itself (61) and in a recent study on the overall cellular metabolism (62). In both studies, transcript levels of individual genes also fluctuated within the pathway, as observed here. However, Cassera *et al.* (61) concluded that, overall, there was no feedback control of transcription by metabolites of the MEP pathway. In plants, in contrast, the MEP pathway is known to be controlled to some extent also on the transcriptional level (63, 64, 65), whereas little data exists for *E. coli* in this respect (54, 66). It should be kept in mind that all MEP pathway genes in apicomplexa are nuclear-encoded but apicoplast-targeted, and continued transcription of numerous of those genes despite apicoplast loss has been recently described in *P. falciparum* (41), indicating a lack of direct communication. Furthermore, treatment of the plant *Catharanthus roseus* with two different MEP pathway inhibitors led to different transcriptional responses of analyzed genes, whereas DXS inhibition by clomazone resulted in DXS (isoform 2A), DXR, and IspG upregulation over time, in fosmidomycin-treated plants these genes responded with an initial increase followed by a decline in transcripts (65).

All these studies inhibited the MEP pathway either at the first (clomazone) or second (fosmidomycin) step, consequently affecting synthesis of downstream metabolites. In contrast, TgFd depletion as shown here directly affects only the last two enzymes, which could potentially result in product accumulation upstream of HMBPP. Therefore, different transcriptional responses between these two regimens could be expected. Whether the very modest transcriptional changes upon TgFd depletion we observe is indicative of an increased dependence on posttranscriptional regulatory mechanisms needs to be studied. Unfortunately, fosmidomycin inhibition with pharmacological concentrations is not possible in *T. gondii*, as noted above, ruling out direct comparisons. Collectively, transcriptional regulation of the MEP pathway upon disturbances of individual metabolites is difficult to conceptualize for different organisms and circumstances.

The phenotype in gliding motility we observed, although moderate, is consistent with the role prenylated and N/C-glycosylated proteins play in this process and which rely directly or indirectly on polyprenols. The small GTPase Rab11A, usually geranylgeranylated, was shown recently to be involved in the regulation of extracellular motility (67), and preliminary results indicate changed patterns of proteins reactive with an antiprenyl group-specific antibody in the molecular size range of Rabs (data not shown). Another protein, GAP50, is an essential membrane-anchored protein and required for gliding motility (68) and which contains N-linked glycans (35). The synthesis of N-glycan lipid-linked oligosaccharide precursors in the ER involves dolichol-pyrophosphate (69). Experimental evidence suggests that *T. gondii* can scavenge dolichol-pyrophosphate-oligosaccharides from its host cell and incorporate them into its proteins upon further modification (70). This is in contrast to the blood stage forms of *P. falciparum*, which rely entirely on their own dolichol synthesis because erythrocytes do not show MVA pathway activity (71). Together, this could explain the relatively small decrease in gliding motility we observe upon TgFd depletion. Likewise, dolichol-phosphate mannose is a precursor for the DPY19-type mannosyltransferases, resulting in C-mannosylation of MIC2, a protein involved in cell adhesion (72). The deletion of TgDPY19 results in reduced parasite adhesion, motility, and invasion (73).

*T. gondii* is auxotrophic for cholesterol, one of the products of the host MVA pathway. It therefore does not come as a surprise that infection with tachyzoites of human and mouse cells leads to increased transcription of some of the MVA pathway genes (74, 75, 76), presumably resulting in increased isoprenoid synthesis. This is in contrast to blood stages of *P. falciparum* because erythrocytes contain minimal amounts of functional MVA pathway enzymes, and IPP levels of uninfected red blood cells are also low (8, 77). Our finding of an observable contribution of the host MVA pathway to a dampened iΔFd phenotype is in agreement with previous studies. Atorvastatin treatment was reported to accelerate parasite death caused by impaired apicoplast function, either caused by gene deletion of *T. gondii*’s cytosolic farnesyl diphosphate synthase (34) or by drug treatment affecting the apicoplast’s overall function (30). Notably, parasite death by the so-called delayed death phenotype (78), as observed in these studies, appears to be still faster than what we observed in the iΔFd strain. In preliminary experiments, this strain showed a ‘delay in the delayed death phenotype’, compared with chloramphenicol-treated wt tachyzoites known to also result in this phenomenon (79) (data not shown). The reasons for this are currently unknown but could be because of residual TgFd protein in the iΔFd strain below the detection limits of our methods used but sufficient to prevent faster death.

TgFd is dependent on TgFNR for its function. In the current annotation of *T. gondii*’s genome, there is no other protein identifiable that could serve as an apparent TgFd reductase. TgFNR also modestly responds on the transcriptional level to TgFd’s depletion (Fig. 7), suggesting a functional connection. In contrast, EcFpr’s function in *E. coli* seems to be redundant (80, 81), and EcFldA’s reduction can be achieved by other enzymes. As pointed out in the introduction, there is great structural and functional flexibility in different cellular systems when it comes to the provision of electrons *via* Fd- or Fld-dependent reductases.

We focused in this study on TgFd’s role on the MEP pathway, whereas it is possible that it also regulates the activity of other redox-dependent enzymes in the apicoplast, including subunits of the lipoic acid synthase which is required for lipoic acid synthesis and activity of the PDH and FASII synthesis (Figs. 1 and 9). Recent studies have shown that under sufficiently high exogenous fatty acid supplementation, a functional FASII is dispensable in *T. gondii*, although some growth retardation was observed under these conditions (39, 82). We therefore assume that iΔFd’s death phenotype is caused mostly by its impact on the two MEP enzymes. Its central role in this essential pathway define apicoplast-resident Fd as a potential drug target in *T. gondii* and other Apicomplexa (19).

## Experimental procedures

### Cells and parasite culture; transfection and generation of stable clones; plaque assay

The strains RHΔHXGPRTΔKu80 (83) and RH-Rep1.2 (expressing TetR^S^; (23)), were kindly provided by D. Soldati-Favre. BJ-5ta human foreskin fibroblasts (HFF; ATCC CRL-4001) were used as host cells, using DMEM (high glucose) plus 10%, 2%, or 1% fetal bovine serum (FBS; Gibco), respectively, called D10, D2, or D1 medium. Parasite and host cell handling, culture conditions, transfection, and cell cloning protocols followed standard procedures (84) unless otherwise indicated.

For the generation of p5RT70TetO-based clones, 1∗10^7^ parasites of strain RH-Rep1.2 were electroporated using a BioRad Gene Pulser II electroporator. Two hours after transfection with 50 μg of p5RT70TetOTP-HA-TgFNR_wt_ or p5RT70TetOTP-HA-TgFNR_S267R_ mutant together with 5 μg pDHFR-TSc3 (85) and subsequent infection of HFF with the electroporated cells, the medium was supplemented with 1 μM pyrimethamine for the selection of resistant transfectants. Once a stable pyrimethamine-resistant population was established, single clones were obtained by limiting dilution and selected for inducible FNR expression (induced by the addition of 0.75 μg/ml aTc) (Cayman Chemical) for 8 h by IFA using an anti-HA antibody (see Table S1).

For the transfection of piKO-based constructs, 6∗10^6^ parasites were electroporated using an Amaxa Nucleofector II Device in Cytomix (program T-016; Lonza). The parasite suspension was transferred to culture flasks with confluent BJ-5ta cells. For the generation of stable clones based on RHΔKU80ΔHXGPRT, 6 h after transfection, the medium was exchanged for DMEM (2% FBS) containing mycophenolic acid (25 μg/ml; Calbiochem) and xanthine (50 μg/ml; Sigma). Subsequently, the parasites were released from host cells by passing them through a 25G needle attached to a syringe (“syringe-released”) every 2 to 3 days and regrown in a new T25 cell culture flask containing selection medium until drug-resistant parasites emerged. For stable complementation of iΔFd::Fd, 2 days after transfection parasites from a T25 flask were added to a new confluent T75 flask. One day later, the medium was exchanged for DMEM containing 2% FBS and 5 μM 5-fluoro-2′-deoxyuridine (Alfa Aesar GmbH). Twenty-four hours later, the cells were syringe-released from the host cells and added to a new T75 flask with confluent cells and fresh drug selection medium. This procedure was repeated twice, each time after 2 days before the single clones were generated by limiting dilution (84). Correct integrations/gene replacements were checked by analytical PCR using appropriate primers as indicated in the figures.

The performance of plaque assays measuring parasite growth over several lytic cycles in the presence of aTc (0.6 μg/ml in ethanol) or absence (ethanol only) has been described by us in detail previously (27).

### Plasmid constructs

For inducible repression of TgFd transcripts, we started with plasmid p5′COR-T8TATi1-HX-tetS1mycNtCOR described in (22). The flanking regions of the coronin gene in this plasmid were excised and replaced by oligonucleotides 17/18 and 19/20 (see Table S2 for all primer sequences), inserting unique PmeI (3′UTR) and SwaI (5′UTR) restriction sites, respectively, resulting in the plasmid piKO1x. The flanking sequences, amplified with Phusion polymerase from either genomic DNA of RH strain (1 kb of 3′UTR of TgFd; primers 21/22) or from cosmid PSBM821 DNA (0.95 kb of TgFd 5′UTR; primers 23/24), were cloned *via* Circular Polymerase Extension Cloning (86) with compatible overhangs into PmeI and then SwaI-cut piKO1x vector, resulting in piKO1x-3/5UTR. Finally, piKO-TgFd was constructed by inserting TgFd, amplified from cosmid PSBM82 DNA with primer 25/26, into EcoRV-cut piKO1x-3/5UTR. The complementation plasmid pGRA-UPRT-TgFd was based on pGRA-GFP-UPRT (26) into which the TgFd gene including 1 kb of 5′UTR sequences, amplified from cosmid PSBM82 with primers 27/28, were cloned *via* homologous recombination into the SmaI site of the plasmid.

The plasmids p5RT70TetO4 TP-HA-TgFNR_wt_ and p5RT70TetO4 TP-HA-TgFNR_S267R_ were based on the plasmid p5RT70TetO4 myc-GFP (23). A fragment encompassing the bipartite apicoplast targeting sequence from TgFNR (aa 1–149; (87)) was amplified from the plasmid p133 (18) using primers 29/30 and inserted into EcoRI/PstI digested p5RT70TetO4-mycGFP. The resulting plasmid was MfeI/XhoI-digested to release mycGFP and substituted by an EcoRI fragment containing the mature part of TgFNR (aa 150–497) from either plasmid pB42AD-FNR_wt_ or pB42AD-FNR_S267R_, respectively (88). Upon import into the apicoplast, it results in an N-terminally HA-tagged mature FNR protein.

All the constructs were verified by sequencing across the cloning sites. All the enzymes used were from NEB.

### Immunofluorescence assays and microscopy

Commercial primary and secondary antibodies and the dilutions used are described in Table S2. Rabbit anti-TgFd antiserum was generated exactly, as described previously for TgFNR (18) using recombinant 6His-TgFd (89). The parasites grown in BJ-5ta on coverslips for 24 to 36 h were processed for IFA, as detailed in (38) and analyzed using a Zeiss Axio Imager Z1/Apotome microscope equipped with a Zeiss AxioCam MRm camera. Image acquisition was done with AxioVision software and processed using equal linear adjustments for all samples. The images in Fig. S2 were acquired with a Leica TCS SP2 confocal laser scan microscope using Leica LCS software.

For correlative light and electron microscopy of plaques BJ-5ta, the cells were grown in μ-dish cell culture dishes (ibidi) to confluency and then infected with 200 parasites of each strain in 3 ml DMEM (2% FBS, +aTc 0.6 μg/ml). The dishes were then incubated undisturbed for 7 days at 37 °C before the cell lawn was fixed and processed for correlative light and electron microscopy exactly, as described previously by us (27).

### SDS-PAGE and immunoblotting

Separation of proteins by SDS-PAGE and subsequent immunoblotting followed standard protocols, as described previously (18).

### Quantitative RT-PCR

The RNA from egressed tachyzoites was purified with the RNeasy Plus Mini Kit (Qiagen) according to the manufacturer's instructions. RNA was converted to cDNA using the Primescript RT-PCR Kit (Takara). Specific primers for different MEV genes were designed using the NCBI primer tool (primers listed in Table S2). Per sample, 10 ng cDNA were mixed with the Luna Universal qPCR master mix (NEB) and respective primers and the reaction performed in a BioRad C1000/CFX96 system and analyzed using CFX Maestro software. The transcript levels were calculated with the ΔΔCt method and expressed as relative expression of transcripts compared with those of the housekeeping gene actin.

### Targeted metabolomics

#### MEP

*T. gondii* strains were cultivated in DMEM +2% FBS in the presence of 0.6 μg/ml aTc for 48 h before infection of ten T150 dishes of confluent BJ5ta cells with ∼3∗10^7^ parasites/dish. One day after infection, the medium was exchanged (+0.6 μg/ml aTc) and intracellular parasites were harvested ∼48 h after infection. The dishes were put on ice, then medium was aspirated before the cells were scraped in a small amount of ice-cold PBS and collected in a 50 ml falcon tube. The parasites were syringe-released from host cells, centrifuged at 1200*g* or 10 min at 1 °C, the pellet was resuspended in ice-cold PBS, and tachyzoites purified *via* filtration through a 3 μm polycarbonate filter into a pre-chilled 50 ml tube. After another centrifugation step at 1200*g* for 10 min, the parasites were washed once with ice-cold PBS and counted using a hemocytometer. 1∗10^9^ parasites per sample were quenched and washed with ice-cold PBS. The pellet was then snap-frozen in liquid nitrogen and stored at −70 °C until all the replicates were collected to perform the metabolite extraction. To this end, 1 ml of 0.1% formic acid in acetonitrile/methanol/water (40:40:20) was added to each sample, after 2 min ultrasonication (10 s pause every 10 s), 1 h incubation at −20 °C, and centrifugation at 21,500*g* for 5 min at 0 °C. The supernatants were purified with Supelclean LC-NH2 solid phase extraction columns (SUPELCO) (32) and eluted with 100 μl 1% ammonia in water. The analytes were chromatographically separated with a 6 min isocratic run on a SeQuant ZIC-pHILIC 5 μm polymer 150 × 4.6 mm (Merck) column with an OPTI-LYNX ZIC-pHILIC 2.1 mm × 15 mm guard column cartridge (Optimize Technologies) and 10 mM ammonium carbonate, 118.4 mM ammonium hydroxide, and 60% acetonitrile in water as a mobile phase. The analytes were measured with an Orbitrap Q Exactive Plus mass spectrometer (Thermo Fisher Scientific) in negative tSIM mode at a resolution of 70,000 and an inclusion list of the following masses: 213.0164, 215.0321, 260.9929, 276.9879, and 244.9980. The chromatographic peak intensities were determined with the QualBrowser (part of the XCalibur package from Thermo Fisher Scientific).

#### Fatty acids

iΔFd was cultured for 2 days in D1 medium (±0.6 μg/ml aTc) with a multiplicity of infection (MOI) of 4.4 in an incubator at 37 °C and 5% CO_2_. After passaging the tachyzoites and re-infecting HFF cells with an MOI of 4.4, the untreated tachyzoites were incubated again in D1 medium (±0.6 μg/ml aTc) for 15 h and the aTc-pretreated ones for 20.5 h before a medium change to ^13^C-D1 medium. After another 24 h, the cells were cooled on ice for 10 min and the parasites were subsequently counted. The undiluted parasite suspension was centrifuged at 300*g* for 20 min at 0 °C, the supernatant was removed, and the pellet was resuspended in 10 ml of ice-cold PBS. Centrifugation was repeated two more times and finally the parasite suspension, each containing 1∗10^8^ parasites, was added to three 1.5 ml reaction tubes. This was followed by centrifugation at 21,500*g* for 1 min at 0 °C, after which the supernatant was removed and 100 μl chloroform was added. The pellet was resuspended and tubes were placed in an ultrasonic bath for 20 s before 400 μl of a 3:1 v/v methanol-ethanol mixture were added and vortex mixed. The solutions were centrifuged at 10,000*g* for 10 min at 4 °C, and the resulting monophasic supernatant was transferred to new 1.5 ml reaction tube containing 100 μl ddwater. The phases were separated by centrifugation and the organic phase was dried using a SpeedVac concentrator, sealed with Parafilm, and stored at −20 °C. Sample preparation and GC-MS analysis was performed, as described (90). Apolar metabolites were subjected to methanolysis in 0.5 M methanolic HCl at 80 °C for 4 h. Free FAs were derivatized in 1% trimethysilyl (BSTFA-1%) for 1 h at RT before the samples were finally analyzed on a DB-5MS plus DG column (30 m × 0.25 mm, 10 gap) on Agilent 7890A-5975C GC-MS. Chromatograms were processed using MSD Chemstation D.01.02.16 software (Agilent Technologies).

### Measurement of trail lengths

100 μl 50% FBS in DMEM was added to each cavity of an 8-well chamber slide, incubated for 2 h at 37 °C, and then rinsed three times with PBS. *T. gondii* strains (iΔFd, iΔFd::Fd and RHΔHXΔKU as wt strain), cultivated with an MOI of 4.4 for 4 days (2 + 2 days) in DMEM (2% FBS, ±0.6 μg/ml), were counted, and 400 μl of the corresponding parasite suspension (1∗10^7^ parasites/ml each) were added to each well, left for 5 min at RT, and then incubated for 15 min in a CO_2_-incubator at 37 °C. After carefully removing, the suspension parasites were fixed with 400 μl 4% paraformaldehyde in PBS for 20 min at RT. After blocking with 400 μl 3% bovine serum albumin in PBS slides were handled as described for IFA, with mouse α-SAG1 monoclonal antibody (1:1000) as primary and goat α-mouse Alexa Fluor 546 (1:4000) as secondary antibody, respectively. The slides were mounted with Fluoromount and imaged as described above. Further processing was performed with ImageJ 1.48v. Trail lengths were measured with the help of the ImageJ plugin NeuronJ (91) using default settings. We regarded as trails more than four stained and evenly spaced spots that could be connected by nonzigzag lines, irrespective of the close presence of tachyzoites (because these are frequently lost during washing steps). To exclude bias during analysis, all images were first randomly arranged and renamed using ImageJ. After all the measurements were completed, original file names were restored. A total of at least 500 trails per strain and condition from three biological replicates were measured and the median length expressed in μm after conversion in ImageJ of pixels into μm.

### Growth assay

To evaluate the growth of *T. gondii* strains under Atorvastatin treatment, the number of parasites per vacuole 30 h after infection were determined by counting as follows. The strains (iΔFd and iΔFd::Fd) were cultured for two passages (96 h) plus/minus aTc (0.6 μg/ml) before fibroblasts grown on coverslips were infected with naturally egressed tachyzoites, then grown for an additional 30 h and subsequently processed for IFA and image acquisition performed, as described above. In some cultures, atorvastatin (Ca-salt; Cayman Chemical) was present at 13 μM during the whole 126 h culture period (added fresh with each medium change). The individual tachyzoites in blinded images were identified by nuclear DAPI stain, whereas parasitophorous vacuoles were outlined by mouse anti-GRA7 staining. For each condition about 50 vacuoles were examined.

### Statistics and software

Data analysis and presentation was performed as indicated in the figures, with Prism 9 (GraphPad), R package ggpubr 0.4.0., or PAST4.3 (92). Chemical formulae were obtained from PubChem and drawn using MarvinSketch (ChemAxon). Image analysis was done with current versions of ImageJ/Fijj.

## Data availability

All data are contained within the article. The material described is available upon request from the corresponding author.

## Supporting information

This article contains supporting information (19, 88, 93, 94, 95, 96).

## Conflict of interest

The authors declare that they have no conflicts of interest with the contents of this article.
