# Supplementary material for: Toxoplasma gondii apicoplast-resident ferredoxin is an essential electron transfer protein for the MEP isoprenoid-biosynthetic pathway
Source: J Biol Chem. 2021 Dec 8;298(1):101468. doi: 10.1016/j.jbc.2021.101468 (PMC8717598; doi:10.1016/j.jbc.2021.101468)
Supplement: Supplemental Figures S1, S2, Tables S1 and S2 [file mmc1.pdf]

Supporting information

Toxoplasma gondii apicoplast-resident ferredoxin is an essential electron transfer protein for the MEP isoprenoid biosynthetic pathway

Stephanie Henkel, Nora Frohnecke, Deborah Maus, Malcolm J. McConville, Michael Laue, Martin Blume, Frank Seeber

Figure S1: Comparison of MVA and MEP pathways for isoprenoid biosynthesis

Supporting results and discussion related to Figure S2 - Attempts to deplete electron flow in the apicoplast by conditional overexpression of an inactive TgFNR enzyme

Figure S2: Stable clones conditionally overexpressing wild-type and mutant *Toxoplasma gondii* ferredoxin-NADP(H) reductase (TgFNR$_{wt}$ or TgFNR$_{S267R}$)

Table S1: Antibodies used

Table S2: Primers used

References

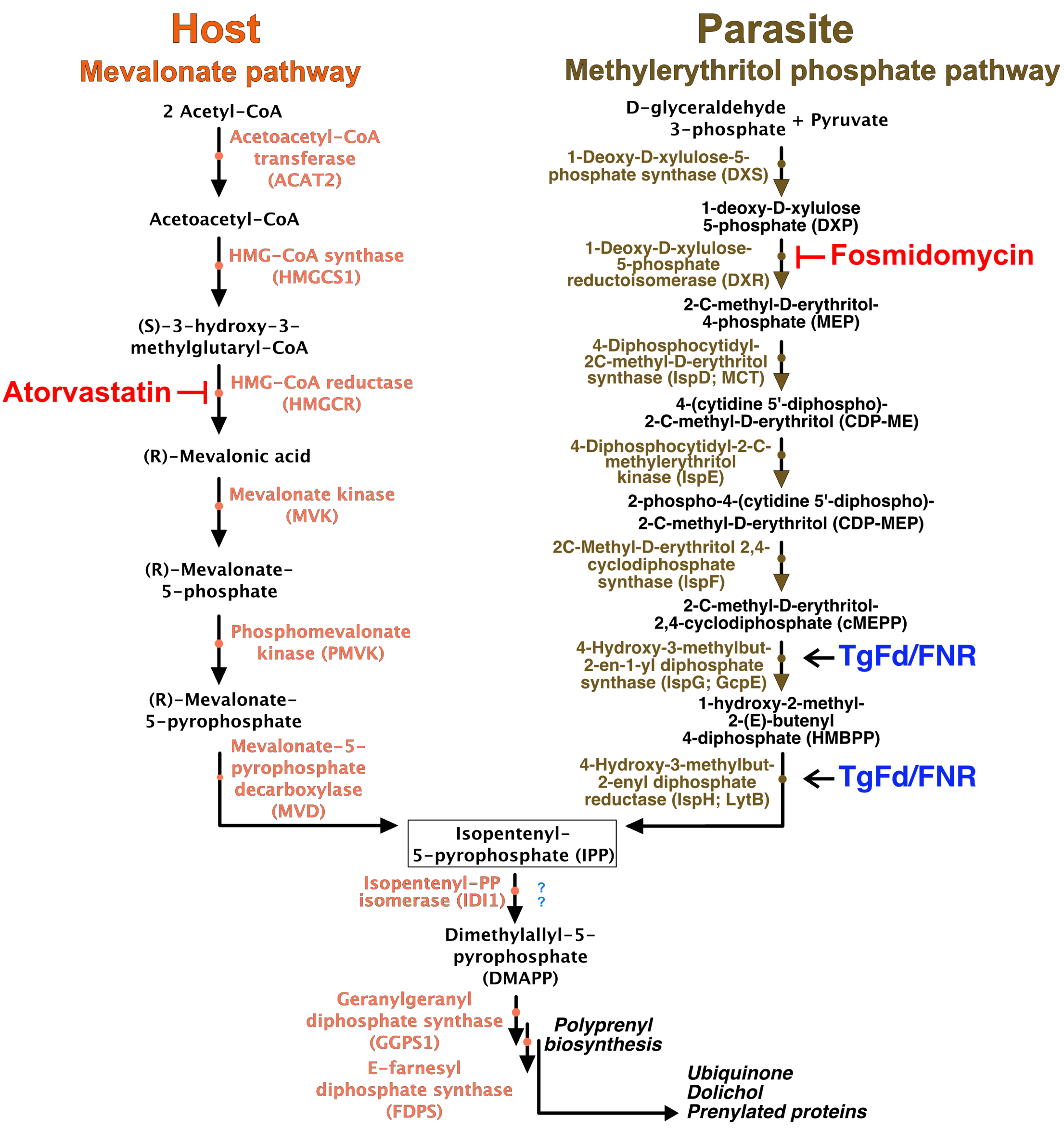

Figure S1: Comparison of MVA and MEP pathways for isoprenoid biosynthesis

Supporting results and discussion related to Figure S2 -

Attempts to deplete electron flow in the apicoplast by conditional overexpression of an inactive TgFNR enzyme

We previously identified a single point mutation (S267R) in the reductase, TgFNR ($TgFNR_{S267R}$), which results in loss of activity and causes a tenfold increase in its affinity for TgFd (1). Based on these properties we reasoned that inducible overexpression of $TgFNR_{S267R}$ in tachyzoites would function as a conditional trans-dominant negative regulator of TgFd function (2). $TgFNR_{S267R}$ is expected to reduce overall electron flow from NADPH to TgFd since it could outcompete the wild-type protein ($TgFNR_{wt}$) and impact TgFd's function as electron carrier by the mutant's higher binding affinity and lack of enzymatic activity. It was hypothesized that this system would allow the tunable suppression of TgFd function which would allow time-resolved metabolomic analyses of dying parasites.

After several attempts we established a stable clone of $TgFNR_{S267R}$ -expressing RH strain parasites that showed correct apicoplast localization (Fig. S2A, left) and tight inducibility upon addition of aTc (Fig. S2B). Although IFA signals pointed to a lower expression level of $TgFNR_{S267R}$ compared to a clone that overexpressed $TgFNR_{wt}$, this was not evident on immunoblots (Fig. S2B). Overexpression and correct folding of the transgene was also apparent by an intense yellow color of cell pellets of both transgenic lines when compared to non-transfected tachyzoites (data not shown), presumably to the much higher amount of TgFNR-bound FAD in these cells. However, despite these facts we could not observe a significant difference in growth under continued inducing conditions during routine culture (data not shown), ruling out that we missed an effect on growth due to a delayed death effect. We therefore did not follow this approach further and turned our attention to the conditional disruption of TgFd's function.

Given the successful use of dominant-negative mutants for protein function impairment in *T. gondii* (3) our failure to see an effect on parasite growth upon overexpression of the inactive $TgFNR_{S267R}$ protein was unexpected, given the strong in vitro phenotype (3% activity left for donating electrons to TgFd; (1)). Whether this low activity together with the tenfold higher affinity towards TgFd results in higher catalytic efficiency which in turn is sufficient to counteract the overexpression of the mutant TgFNR, thus preventing a negative phenotype,

needs to be determined. Dominant-negative effects of a mutant $NADP^+$-dependent enzyme, isocitrate dehydrogenase, could also not be observed upon overexpression in human cells, although it was anticipated from in vitro data (4).

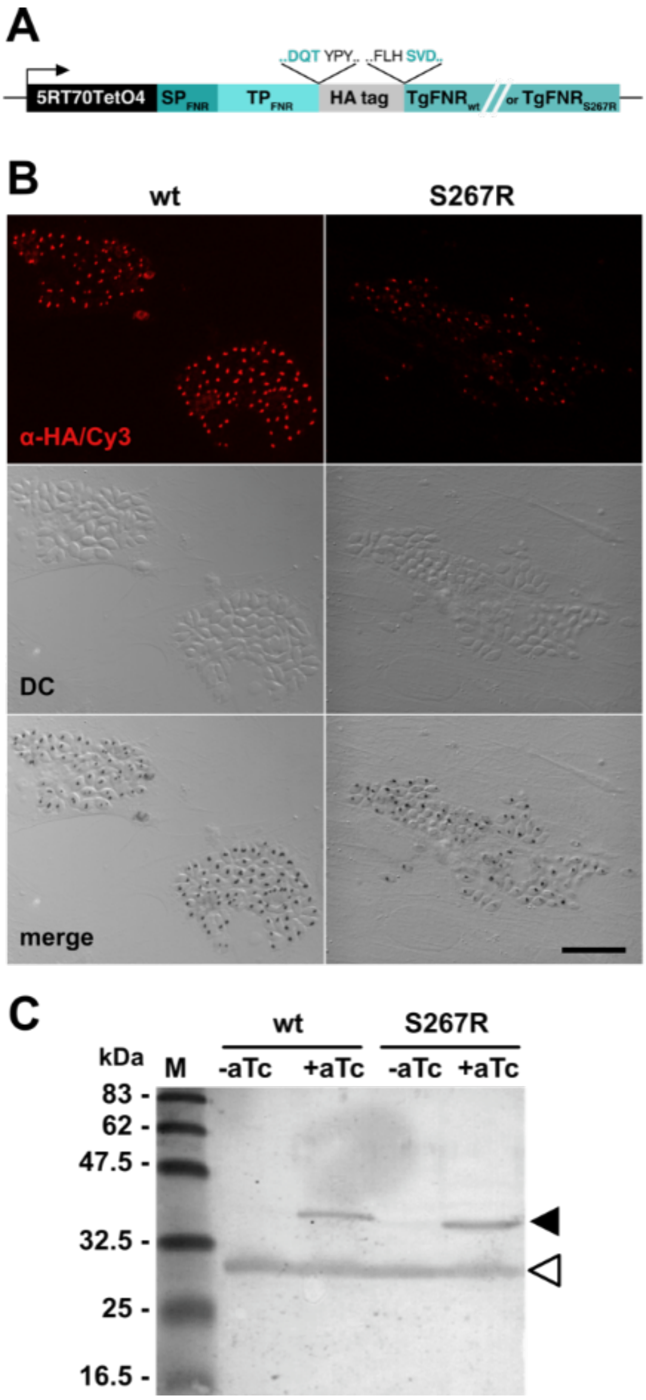

Fig. S2 Stable clones conditionally overexpressing wild-type and mutant *Toxoplasma gondii* ferredoxin-NADP(H) reductase ($TgFNR_{wt}$ or $TgFNR_{S267R}$). **A.** Scheme of construct used for overexpression. **B.** Indirect immunofluorescence microscopy of HFF cells infected with *T. gondii* tachyzoites. Fluorescence shows N-terminally tagged hemagglutinin FNR fusion protein. The scale bar represents 50 µm. **C.** Western blot analysis with anti-HA antibodies for

detection of epitope-tagged FNR. In B only aTc-induced cultures are shown. Scale bar = 20 µm. In C SAG1 (open arrow head) served as loading control, detected with monoclonal antibody DG52. The observable slightly faster migration of $TgFNR_{S267R}$ (solid arrow head) is due to the additional arginine residue in this mutant (1).

Table S1: Antibodies used

**Antibodies**	**Dilutions**	**Source**
Mouse α-cMyc mAb (clone 9E10)	1:4,000	Santa Cruz Biotechnology, Dallas, TX, USA
Rabbit α-Fd pAb	1:500	(5)
Rat α-HA mAb (clone 3F10)	1:100	Roche Diagnostics GmbH, Berlin, D
Mouse α-SAG1 mAb (clone DG52)	1:1,000	(6)
Streptavidin-Cy5	1:2,500	Jackson ImmunoResearch Europe Ltd, Suffolk, UK
Streptavidin-Alexa Fluor® 488	1:4,000	Thermo Fisher Scientific Inc., Karlsruhe, D
Mouse α-SAG1 mAb (clone DG52)	1:1,000	Thermo Fisher Scientific Inc., Karlsruhe, D
Goat-α-mouse pAb Alexa Fluor® 546	1:4,000	Thermo Fisher Scientific Inc., Karlsruhe, D
Goat-α-mouse pAb Dylight® 650	1:1,000	Agrisera AB, Vännäs, Schweden
Goat-α-rat pAb Alexa Fluor® 488	1:1,000	Thermo Fisher Scientific Inc., Karlsruhe, D
Goat-α-rat pAb Cy3	1:300	Jackson ImmunoResearch Europe Ltd, Suffolk, UK
Goat-α-rabbit pAb HRPO	1:1,000	Jackson ImmunoResearch Europe Ltd, Suffolk, UK

Table S2: Primers used

Primer number (gene ID)	Primer name	Primer sequence (5' > 3')
17	piKO1x-SbfISwaIFW	CATGGCCTGCAGGATTTAAATG
18	piKO1x-SbfISwaIBW	GATCCATTTAAATCCTGCAGGC
19	piKo1x-PmeIFW	GATCTGTTTAAACA
20	piKO1x-PmeIBW	CTAGTGTTTAAACA
21	3-piKO-3UTR_CP	CTGCAGGCGGCCGCGAATTCACTAGTGTTTCCTGCGACGACACACGAG
22	5-piKO-3UTR_CP	GCTCATCTCCGAGGAGGACCTGAGATCTGTTTGAGCGCCTGCTCTCGCAAGC
23	3-piKO-5UTR_CP	GTCGAGGGGGGGCCCGGATCCATTTCCGTGCTGTCTCCGCAGC
24	5-piKO-5UTR_CP	CCGGCCGCCATGGCCTGCAGGATTTCCGGCGTCTGGCGACAAACG
25	3-piKO-Fd_CPEC	GAGATGAGCTTCTGCTCACTGCCACTGCCCTCGTCTCCGCCTTCACT
26	5-piKO-Fd_CPEC	CTTCATTATTTCTTCTGGTTTTTTGACGAGTGAATTCCTAGGGATATCATGGCGGACGCCTCCCTG
27	5'-endPUPRT_SLIC	GTGGCGCGCCCGGTCCGCCCGAGTTTGAGAATGTGAGGGG
28	3'-FdUPRT_SLIC	GGGACGTCGTACGGGTACCCCTCGTCTCCGCCTTCACT
29	TP-HA3’	TGCATGCATTTAATTAACAATTGCTGCAGAGCGTAG
30	TP-HA5’	GCGAATTCCCTTTTTCGACAAAATGGTTCGGGGCAT
31 (XM_018779434.1)	DXS_q2FW	CGTGAGATGGAAGGGGCAT
32	DXS_q2REV	GAGACGTTCCTCGACTGCTTTCTA
33 (XM_018779664.1)	DXR_q2FW	ATTTTGTGCTGTCCGGTGTC
34	DXR_q2REV	CAGCAAAACCGTCGAGACAAG
35 (XM_002364846.2)	IspF_q2FW	GCAGGTGAAAGGAGACCAAC
36	IspF_q2REV	ATGACAAGGGGACCTGTAGCC
37 (XM_002365304.1)	IspG_q2FW	GACTTGCCAGCTCTTTCCCT
38	IspG_q2REV	TTTCCGCCGAGACTTTCTGA
39 (XM_002366344.2)	IspH_q2FW	TCGTTGCTTCTTTCCAAGCC
40	IspH_q2REV	GCCCTTTTTGCGCATGG
41 (XM_018782355.1)	FNR_q2FW	ACCGACCAAACATCCGTTGA
42	FNR_q2REV	CGAAGCCGGACGAAAGGTAT
43 (XM_002370787.2)	Fd_qFW	GCAGACCCCCGACGGGGAAACC
44	Fd_qREV	GCCCATCAACAGCTTGCCTGCGC

45 (XM_002369622.2)	Tg_actin-fwd	GCGCGACATCAAGGAGAAGC
46	Tg_actin-rev	CATCGGGCAATTCATAGGAC

References

1. Thomsen-Zieger, N., Pandini, V., Caprini, G., Aliverti, A., Cramer, J., Selzer, P. M., Zanetti, G., and Seeber, F. (2004) A single in vivo-selected point mutation in the active center of *Toxoplasma gondii* ferredoxin-NADP$^+$ reductase leads to an inactive enzyme with greatly enhanced affinity for ferredoxin. *FEBS Lett* **576**, 375-380
2. Herskowitz, I. (1987) Functional inactivation of genes by dominant negative mutations. *Nature* **329**, 219-222
3. Jiménez-Ruiz, E., Wong, E. H., Pall, G. S., and Meissner, M. (2014) Advantages and disadvantages of conditional systems for characterization of essential genes in *Toxoplasma gondii*. *Parasitology* **141**, 1390-1398
4. Jin, G., Reitman, Z. J., Spasojevic, I., Batinic-Haberle, I., Yang, J., Schmidt-Kittler, O., Bigner, D. D., and Yan, H. (2011) 2-hydroxyglutarate production, but not dominant negative function, is conferred by glioma-derived NADP-dependent isocitrate dehydrogenase mutations. *PLoS One* **6**, e16812
5. Seeber, F., Aliverti, A., and Zanetti, G. (2005) The plant-type ferredoxin-NADP$^+$ reductase/ferredoxin redox system as a possible drug target against apicomplexan human parasites. *Curr. Pharm. Design* **11**, 3159-3172
6. Burg, J. L., Perelman, D., Kasper, L. H., Ware, P. L., and Boothroyd, J. C. (1988) Molecular analysis of the gene encoding the major surface antigen of *Toxoplasma gondii*. *J. Immunol.* **141**, 3584-3591
